# Leveraging explainable AI for gut microbiome-based colorectal cancer classification

**DOI:** 10.1186/s13059-023-02858-4

**Published:** 2023-02-09

**Authors:** Ryza Rynazal, Kota Fujisawa, Hirotsugu Shiroma, Felix Salim, Sayaka Mizutani, Satoshi Shiba, Shinichi Yachida, Takuji Yamada

**Affiliations:** 1 School of Life Science and Technology, Tokyo Institute of Technology, Tokyo, Japan; 2 Metagen, Inc., Yamagata, Japan; 3 Metagen Theurapeutics, Inc., Yamagata, Japan; 4 Digzyme, Inc., Tokyo, Japan; 5 Division of Genomic Medicine, National Cancer Center Research Institute, Tokyo, Japan; 6 Department of Cancer Genome Informatics, Graduate School of Medicine, Osaka University, Osaka, Japan

## Abstract

Studies have shown a link between colorectal cancer (CRC) and gut microbiome compositions. In these studies, machine learning is used to infer CRC biomarkers using global explanation methods. While these methods allow the identification of bacteria generally correlated with CRC, they fail to recognize species that are only influential for some individuals. In this study, we investigate the potential of Shapley Additive Explanations (SHAP) for a more personalized CRC biomarker identification. Analyses of five independent datasets show that this method can even separate CRC subjects into subgroups with distinct CRC probabilities and bacterial biomarkers.

**Supplementary Information:**

The online version contains supplementary material available at 10.1186/s13059-023-02858-4.

## Background

A growing number of studies have reported a link between the alteration in gut microbiome compositions and colorectal cancer (CRC). The elevated abundance of certain bacterial species such as *Fusobacterium nucleatum* and *Parvimonas micra* in CRC patients is often associated with the development of the disease [1–3]. These findings have motivated the idea of using fecal biomarkers for CRC diagnosis.

To obtain potential CRC biomarkers and to classify CRC vs. healthy groups, researchers have leveraged the potential of machine learning (ML) algorithms. Among various algorithms, random forest is often used in recent microbiome studies [3–5] due to its predictive power and its ability to generate feature importance. The feature importance information is used to find out which bacteria are most correlated with CRC. A species with high feature importance indicates that it has a high contribution in differentiating CRC and healthy groups.

Furthermore, previous studies often used random forest built-in feature importance that is based on the mean decrease in Gini impurity. This kind of feature contribution method is referred to as the global explanation or global feature contribution technique since it explains ML model outcomes by considering the entire input data. Although this method allows us to discover bacteria that are generally correlated with CRC, it fails to recognize species that are only influential for a smaller group of patients. For instance, it is possible that for some patients, certain species of bacteria have a higher contribution in distinguishing healthy vs disease phenotype compared to *F. nucleatum*, *P. micra*, and other widely known CRC-associated bacteria. To obtain such information, it is better to use local explanations instead of global explanations.

Unlike global explanation which considers the whole data inputs, local explanations focus on each individual input. It can generate feature contributions for every single ML prediction. In other words, local explanation techniques make it possible to discover the most contributing bacteria for each person. Moreover, this will also allow us to examine inter-individual differences between subjects within the disease group. As a result, we will be able to further classify the disease group into subgroups of CRC patients based on the contribution of each bacterial species to the classifier.

Despite the advantages mentioned above, the current studies in the microbiome community have not explored the potential of local explanation-based techniques. Furthermore, to our knowledge, there is no tool that allows researchers to use these techniques for microbiome data analyses.

In this study, we investigated the potential of using a local explanation technique called the Shapley Additive Explanations (SHAP) for gut microbiome data analyses. We explored the advantages of using SHAP for individual feature contribution analyses, principal component analyses (PCA), and CRC subtyping. In the end, we also created a python library to help microbiome researchers perform similar analyses. We believe that this method will be beneficial for the microbiome community and will encourage researchers to leverage local explanations for a more personalized feature importance identification and phenotype subtyping.

## Results

### Local explanations show different sets of influential bacteria among CRC patients

In our analysis, we included 5 publicly available bacterial abundance datasets from different microbiome studies. To obtain these datasets, we utilized the *curatedMetagenomicData* R package [6]. The information about sample size and country of origin of each dataset is summarized in the following table. From now on, we will refer to each dataset using the dataset name shown in the table.

**Fig. 1:**
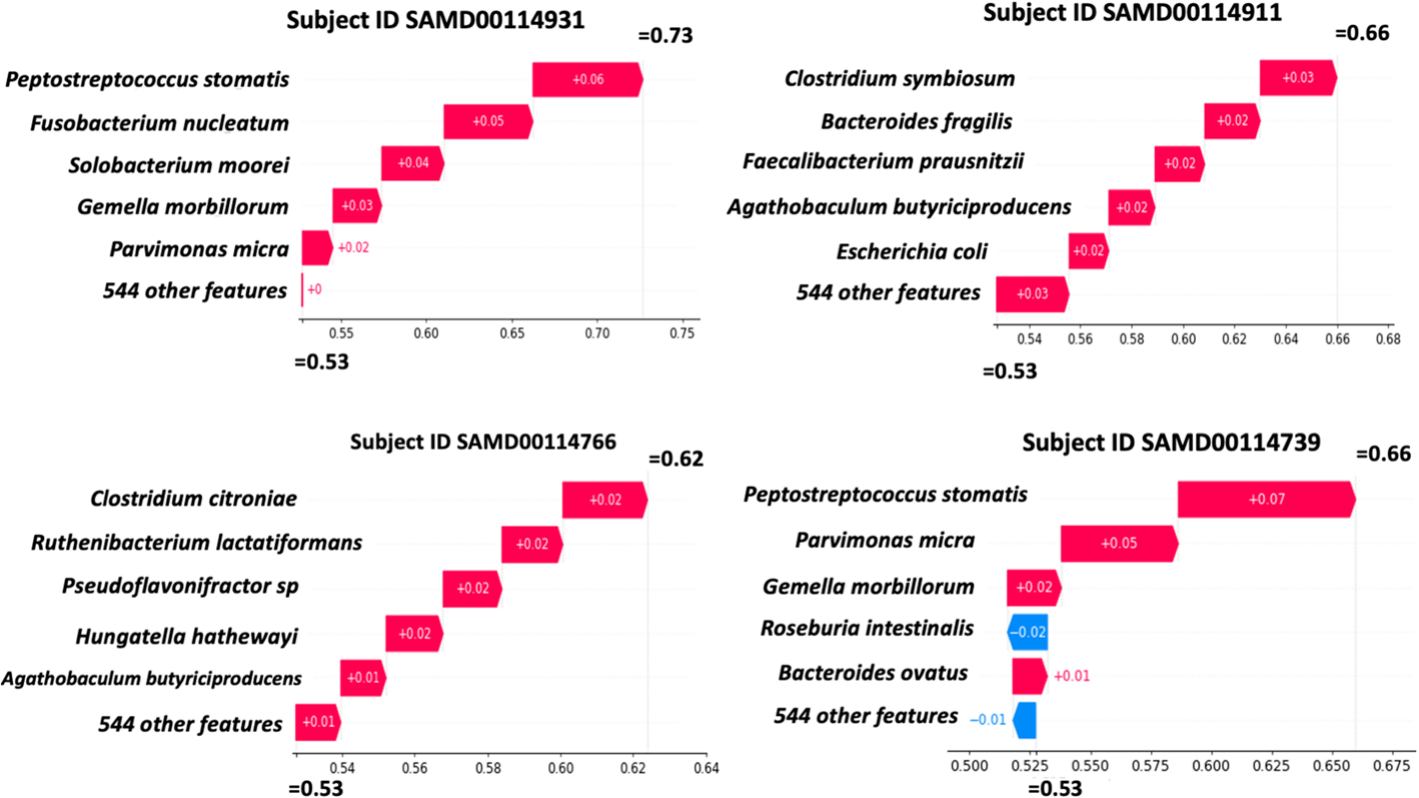
Waterfall plots of local explanations that correspond to four CRC subjects. At the bottom part of each plot, we can see the base value (0.53) which represents the expected value of the CRC class (the mean of model output over the training data). All SHAP values in each local explanation sum up to the predicted CRC probability of each subject (local additivity property of SHAP)

To generate some examples of local explanations, we used the YachidaS_2019 dataset as the test data and the other dataset as the train data. We trained a random forest classifier using the Scikit-learn library [10] on the train data and evaluated the result using the area under the receiver operating characteristic curve (AUC) as the evaluation metric. The result shows a mean AUC of 0.72. Next, we utilized the *TreeExplainer* framework developed by Lundberg et al. [11] to calculate and visualize SHAP values. The local explanations of four randomly chosen CRC subjects from the YachidaS_2019 dataset are shown in Fig. 1.

Each explanation consists of SHAP values that represent the contribution of every feature (bacteria) to a prediction made by the classifier. In each waterfall plot, the SHAP values are ordered vertically from the largest to the smallest values, meaning that the most influential bacteria for a specific person are listed on top. For instance, for the first patient (subject ID: SAMD00114931), *Peptostreptococcus stomatis* has the largest contribution to the classifier prediction, followed by an *F. nucleatum* and *Solobacterium moorei*. For each local explanation, all of its SHAP values sum up to the classifier output (predicted CRC probability). For example, all SHAP values of the first patient add up to 0.73, which is the CRC probability predicted by the classifier. This property is known as local accuracy or additivity [11].

The local explanations allow us to observe different patterns of bacterial contributions among CRC patients. This result cannot be achieved using any global explanation methods including the random forest’s built-in Gini impurity-based method. Using these explanations, we can see that for the patient with subject ID SAMD00114911 in Fig. 1, widely known CRC-associated bacteria such as *F. nucleatum* might not have a high contribution for these specific individuals. Instead, *Clostridium symbiosum* was detected as the most influential bacteria for this subject.

**Fig. 2:**
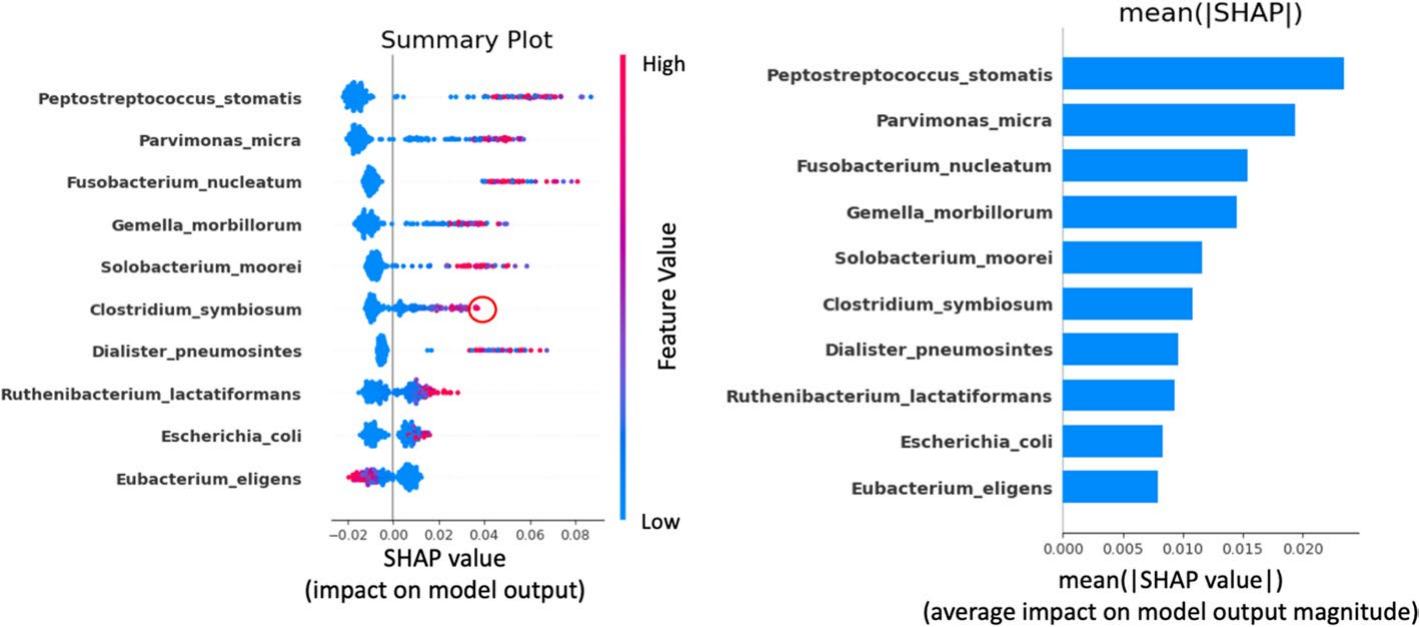
Summary plot (left) and bar chart of global feature importance calculated by taking the mean of absolute SHAP values (right). The summary plot (left) allows us to observe the direction of effects and to discover specific bacteria that have low global feature importance but are very influential for some individuals. The point marked by a red circle on the left represents such bacteria. The bar chart of global feature importance (right) was created by calculating the mean(|SHAP value|) of every bacteria. It shows the average impact of each bacterium on the model output. This bar chart is similar to the global feature importance obtained using the random forest’s Gini impurity-based method. Unlike the summary plot, this bar chart is unable to show the directions of effects or reveal certain bacteria that have low global feature importance but high local feature importance

Moreover, to observe the directions of effects, we plot the SHAP values of all individuals as a set of beeswarm plots known as the summary plot shown in Fig. 2 (left). This plot summarizes the SHAP values of all subjects where each dot represents a value for a subject. The bacteria are ordered from top to bottom based on their mean absolute SHAP values, which correspond to the global feature importance. The *x*-axis position of the dot quantifies the impact that a bacterial species has on the classifier prediction for a specific person. The colors represent the original feature values (relative abundance) where blue and red correspond to low and high abundance, respectively. The figure shows that for the top 9 bacteria, most dots with high relative abundance (colored in red) are located on the positive side of the *x*-axis. This means that a high abundance of these bacteria is associated with a higher probability of CRC. By contrast, most blue dots are accumulated in the negative *x*-axis, showing that lower abundances of these bacteria are associated with a lower probability of CRC. On the other hand, *Eubacterium eligens* shows the opposite pattern, suggesting that a higher abundance of this species is associated with a lower CRC probability and vice versa. This insight into the direction of effects cannot be obtained using widely used global explanation methods such as the random forest’s built-in feature importance.

Furthermore, we can observe that some beeswarm plots have a very long right tail (Fig. 2, left). This shows that bacteria with relatively low global feature importance can still have a high impact on some individuals. For instance, the dot marked by the red circle in Fig. 2 indicates that even though *C. symbiosum* has a relatively low global feature contribution, it still shows a high local contribution to the classifier prediction for this specific person. If we only depend on global explanation methods such as the random forest’s mean decrease in Gini impurity, we will not be able to get such information.

### Analyses of 5 independent datasets show that local explanations lead to more interpretable PCA results

Principal component analysis (PCA) is often used as a dimensionality reduction technique for data visualization and exploration. We compared the results of performing PCA on SHAP values vs. relative abundance data across all datasets. To do this, we first calculated the SHAP values by performing leave-one-dataset-out (LODO) analysis. For instance, in one iteration, we used the YachidaS_2019 dataset as test data and the remaining dataset as train data. For each iteration, we trained a random forest classifier on the train data and measure the AUC using the test data. After that, we finally calculate the SHAP values of the test data using the trained model and an explainer. We did this analysis for every dataset. Please refer to the “Methods” section for a more detailed explanation of the LODO analysis.

**Fig. 3:**
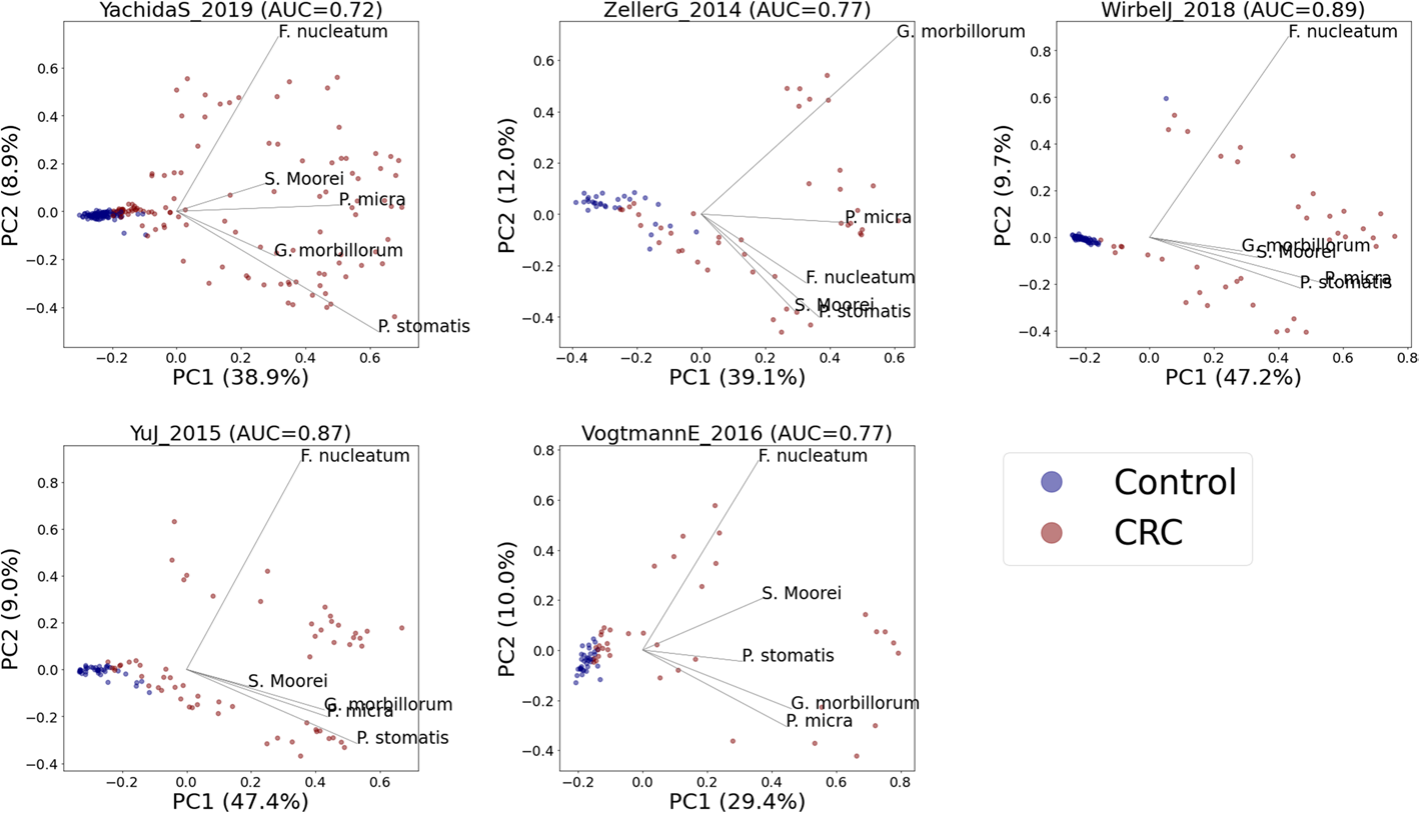
PCA biplot of SHAP values. Blue and brown dots represent the healthy and CRC subjects, respectively. The LODO AUC is shown on the title of each plot. CRC-associated bacteria such as *Fusobacterium nucleatum* (*F. nucleatum*), *Parviomnas micra* (*P. micra*), *Solobacterium moorei* (*S. moorei*), *Peptostreptococcus stomatis* (*P. stomatis*), and *Gemella morbillorum* (*G. morbillorum*) show high PC loadings suggesting their strong correlations to the principal components

**Fig. 4:**
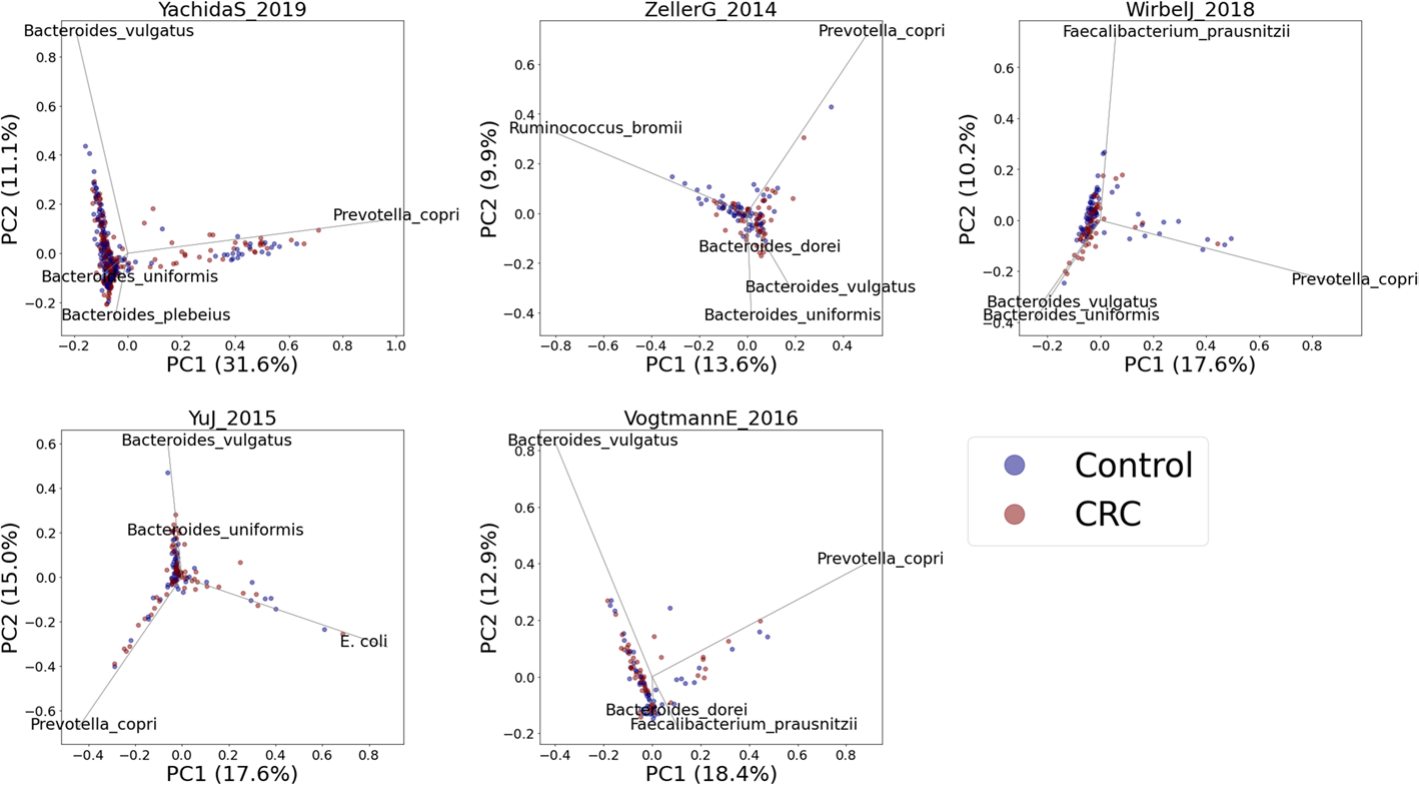
PCA plots of the relative abundance data. Blue and brown dots represent the healthy and CRC subjects, respectively. Unlike in Fig. 3, here, we cannot observe a clear separation between the control and CRC groups

After obtaining the SHAP values, we performed PCA on these values. We chose to calculate the first two PCs (PC1 and PC2), calculated the PC loadings, and visualized the results (Fig. 3). For comparison, the PCA result of the relative abundance data is shown in Fig. 4.

Figure 3 shows the projection of SHAP values onto the first two principal components. Looking at the results from all the datasets, we can consistently observe a clear separation between the healthy and CRC subjects along PC1. By contrast, the PCA results of relative abundance data (Fig. 4) do not show a clear separation between healthy and CRC samples. Moreover, we can observe that relevant CRC-associated bacteria such as *F .nucleatum*, *P. micra*, *Solobacterium moorei*, *Peptostreptococcus stomatis*, and *Gemella morbillorum* show high PCA loadings in the case of SHAP value-based PCA (Fig. 3). This shows that the SHAP value embedding (also called the local explanation embeddings) can capture relevant information to help us observe the variation of interest (healthy vs. CRC) better than the original form of the abundance data.

**Fig. 5:**
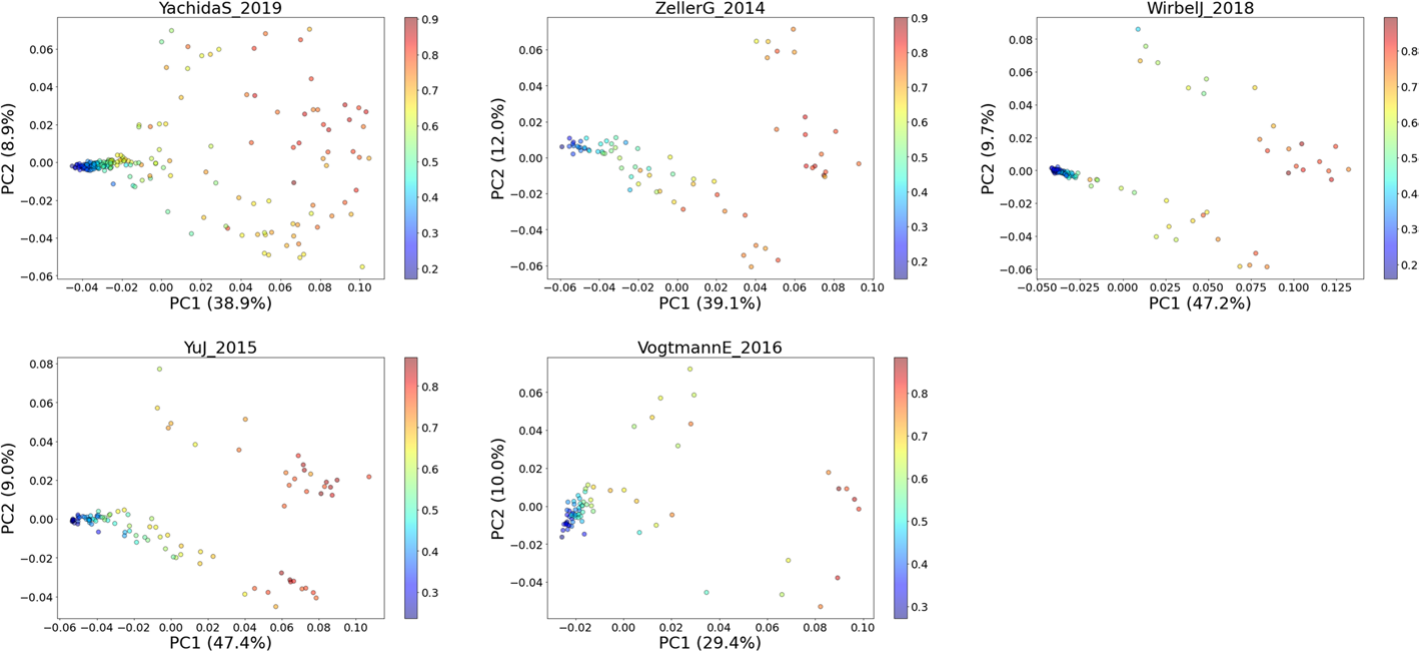
PCA plots of SHAP values overlaid by CRC probability. We can observe a clear trend of increasing CRC probability from the left to the right side of PC1

In addition, we also colored the points on the scatter plot by the predicted CRC probability (Fig. 5) obtained from the classifier output. We can observe a consistent pattern in all datasets in which the individuals located on the right side of PC1 tend to have a higher CRC probability and vice versa. This shows another advantage of applying PCA on SHAP values as it can give us a more interpretable PCA result in terms of the trend in CRC probability across the PC space.

### Local explanation embeddings can be used to cluster CRC subjects

Since there are a clear separation between CRC and healthy subjects (Fig. 3) and a consistent trend of CRC probability (Fig. 5), it is possible to cluster the CRC data points into subgroups of CRC and hypothesize that the resulting subgroups would have similar CRC probabilities. To test this, we performed *K*-means clustering on PC1 and PC2 of the SHAP values that belong to the CRC subjects for each dataset. The number of clusters was chosen using the elbow method (see Additional file 2: Fig. S1).

**Fig. 6:**
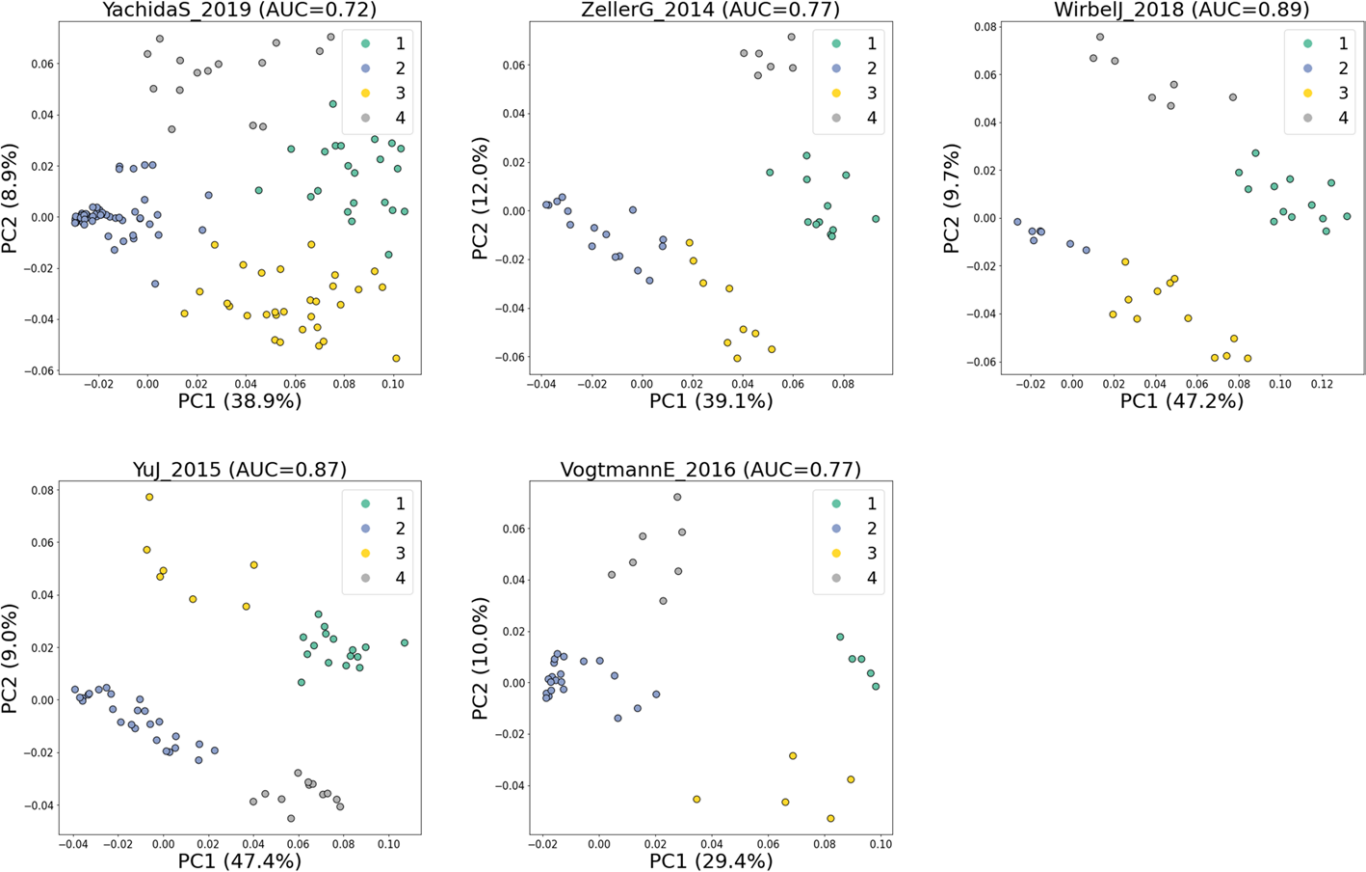
Result of *K*-means clustering on the local explanation embeddings of CRC subjects

**Fig. 7:**
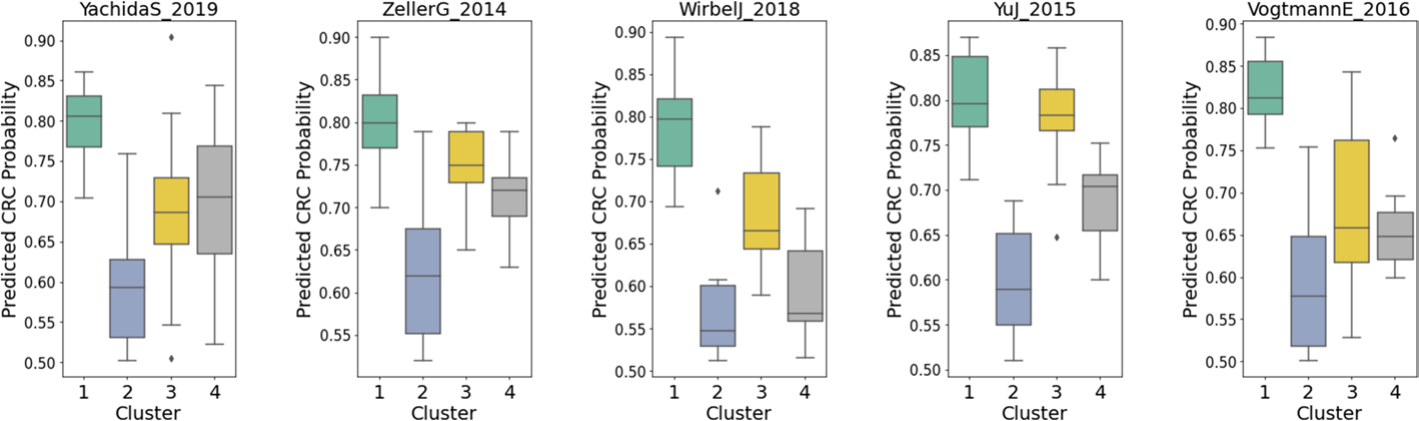
Boxplots showing the distribution of CRC probabilities across different clusters in each dataset

Figure 6 shows the resulting *K*-means clusters of CRC subjects in each dataset. Four clusters were detected in each dataset. As shown in Fig. 7, in each dataset, specific clusters showed a higher CRC probability than the others. CRC subgroups identified as cluster 1 in all datasets show the highest median of CRC probability compared to the rest of the clusters. Similarly, subgroups identified as cluster 2 in every dataset have the lowest CRC probability median. Further statistical analyses show that cluster 1 of all datasets is enriched by *F. nucleatum* compared to healthy subjects and other clusters (see Additional file 2: Figs. S2-S6). This might explain why cluster 1 has the highest CRC probability than the remaining CRC clusters (Fig. 7).

From these results, we can see that this analysis is beneficial for researchers to conduct clustering on the CRC group by focusing on the variation of interest (CRC probability). Using this cluster information, researchers can further conduct statistical analyses to examine dominant bacteria that are associated with each cluster.

### SHAP-based Microbiome Analyses Tool (SHAPMAT)

We created a python library called the SHAP-based Microbiome Analyses Tool (SHAPMAT) to help researchers implement the pipeline that we have presented in this study. This library provides implementations for data preprocessing (abundance and prevalence filtering), SHAP value computation, local explanation visualization, PCA, and clustering. Using this library, microbiome researchers can readily perform a more personalized feature importance identification to find potential CRC biomarkers. In addition, the tool can also be used to get a more interpretable PCA for data exploration and to obtain CRC clusters using local explanation embeddings. Please refer to the “Methods” section for the details of the implementation.

## Discussion

We explored the potential of using local explanations for gut microbiome data analyses, particularly in the context of CRC classification and its potential biomarker identification. Using local explanations, we discovered different patterns of bacterial contributions to the CRC probability among individual CRC subjects (Fig. 1). We also showed that using the summary plot (Fig. 2, left), users can uncover local patterns that are hidden in the dataset that would otherwise be impossible to spot using the existing global explanation methods such as the Gini impurity-based technique.

Moreover, using 5 independent datasets from different microbiome studies, we demonstrated the potential of using SHAP values to generate a more interpretable PCA result for microbiome-disease data exploration. Microbiome data are highly dimensional, where the number of features (taxa) is a lot higher than the total samples [12]. Reducing this data dimension and projecting it onto a lower-dimensional space are very helpful for data exploration and visualization. In particular, PCA is often used for visualizing similarity among samples in a 2D or 3D space and is a useful step prior to the clustering or classification of samples [13]. Unfortunately, PCA results do not always show the variation or trend of interest. This is because the algorithm is designed to find the directions with the largest variation and not the ones that are relevant for separating phenotypes of interest [13]. In our observation, we observe this from the PCA plot of the relative abundance data (Fig. 4). We can see that there is no clear separation between healthy and CRC samples. On the other hand, by performing PCA on SHAP values instead of relative abundance data, we were able to create a plot that shows a clearer separation between healthy and CRC samples (Fig. 3). We can also see the trend of increasing CRC probability along the PC1 (Fig. 5), where samples on the right side of PC1 tend to have a higher probability of getting CRC and vice versa.

Furthermore, we also showed that it was possible to cluster CRC subjects into several subgroups based on the local explanation embeddings. The resulting clusters differ in their CRC probability. Further statistical analyses showed that in all datasets, distinct species of bacteria are enriched in these clusters. For instance, the cluster with the highest CRC probability (cluster 1) in every dataset is always enriched by CRC-associated bacteria such as *F. nucleatum*.

## Conclusion

In summary, we explored the potential of using explainable AI for gut microbiome-based CRC classification. We showed that SHAP could be used to obtain more personalized feature importance that can be used to identify potential bacterial biomarkers for CRC. Our proposed method is also beneficial for data exploration in the context of microbiome-disease association through the generation of interpretable PCA results. In addition, it can also be used to uncover potential CRC subgroups which differ in their CRC probability and associated bacteria. Finally, we also created a software implementation to help microbiome researchers reproduce similar results.

There is still room for improvement. For example, while we only focused on CRC, other types of microbiome-associated diseases such as ulcerative colitis, Chron’s disease, and liver disease [14] can also be analyzed in the same manner using our method. Furthermore, creating a web interface application with the same functionalities is also one of the future works that will benefit the microbiome community by making it even easier to explore the local explanation-based feature importance technique without having to code at all.

## Methods

### Datasets

We used the *curatedMetagenomicData* R package [6] to obtain five independent taxonomic abundance datasets shown in Table 1. The R package uses MetaPhlAn3 to obtain the taxonomic abundance. Only CRC subjects and healthy controls were selected, adenoma samples were not included in the analysis.

**Table 1:** Information about the datasets

Dataset name	Groups (*n*)	Country
YachidaS_2019 [3]	Control (146) CRC (185)	Japan
YuJ_2015 [7]	Control (54) CRC(74)	China
WirbelJ_2019 [5]	Control (65) CRC(60)	Germany
ZellerG_2014 [8]	Control (61) CRC(53)	France
VogtmannE_2016 [9]	Control (52) CRC(52)	USA

### Data filtering

SHAPMAT provides two steps of data filtering: abundance filtering and prevalence filtering. For abundance filtering, users can specify an abundance threshold (default: 10^−15^). Data points that are below this threshold will be set to zeros. In addition, there is also prevalence filtering where users can specify a prevalence threshold (default: 0.9). For each feature (bacteria), if the percentage of the zero abundance is above the prevalence threshold, then it will be removed.

### Leave-one-dataset-out (LODO) analysis

**Fig. 8:**
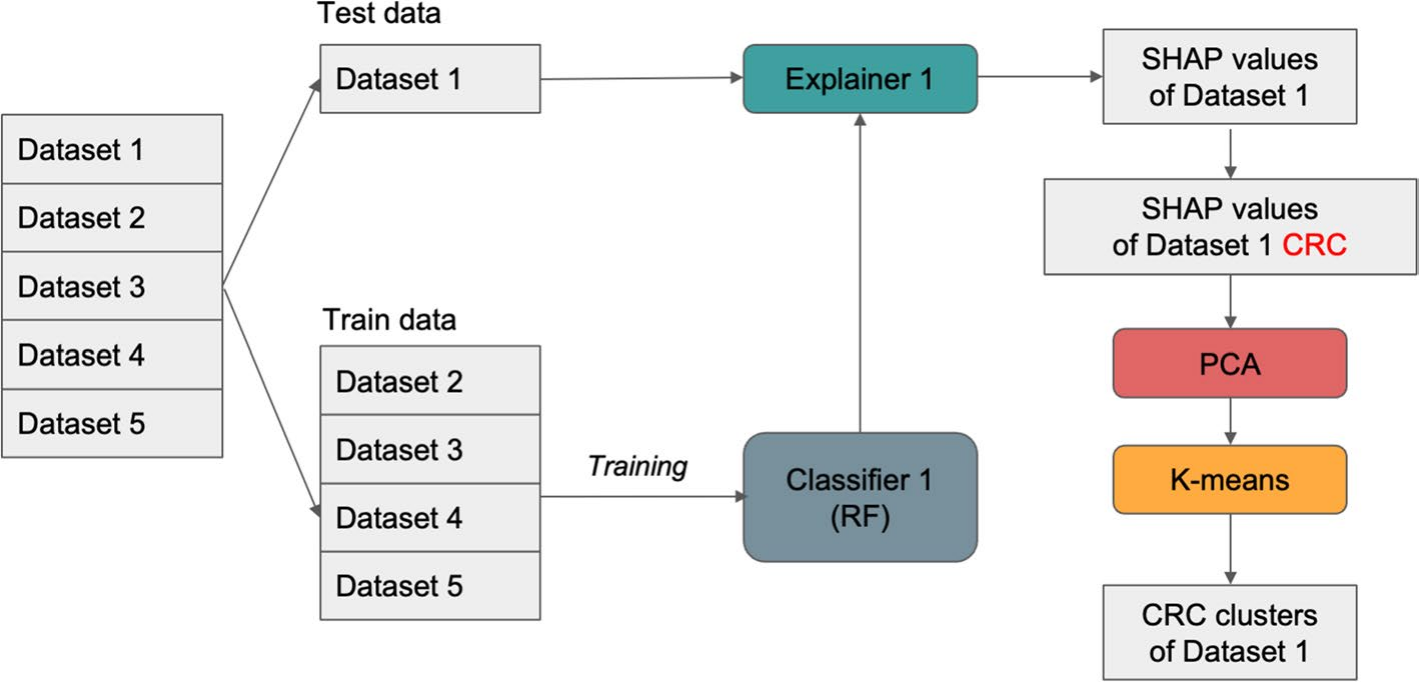
Workflow of the leave-one-out-dataset (LODO) analysis. This diagram shows one iteration of the LODO analysis

The leave-one-dataset-out (LODO) analysis was done to calculate SHAP values for each dataset. For instance, in one iteration, we used the YachidaS_2019 dataset as test data and the remaining dataset as train data. We used the *scikit-learn* [15] library to train a random forest (RF) model and calculated the SHAP values of the test data using the *TreeExplainer* [11]. The input to this explainer includes the test data and the trained RF model. The LODO workflow is shown in Fig. 8.

After obtaining the SHAP values, SHAPMAT provides functionalities for applying PCA and *K*-means clustering. In our analysis, we selected the SHAP values of the correctly predicted CRC subjects. Then, we performed PCA to get the first two principal components and finally cluster the result using k-means.

### Shapley additive explanations (SHAP)

Shapley Additive Explanations (SHAP) is an explainable AI method that is used to explain the output of a machine learning model. This method was developed based on a concept from game theory called the Shapley value. In coalitional game theory, this value tells us how to distribute payout among the players in a coalition, in a fair manner. SHAP applies this idea to explain a machine-learning prediction of an instance by estimating the contribution of each feature to the prediction.

A SHAP value *ϕ*_*i*_ of a feature *i* for a prediction *p* can be defined as follows:

$${\phi}_i(p)=\sum_{S\subseteq N/i}\frac{\mid S\mid !\left(n-|S|-1\right)!}{n!}\left(p\left(S\cup i\right)-p(S)\right)$$

where *n* is the total number of features [16]. Basically, this equation calculates the difference between a model prediction with and without feature *i* in the coalition *S*.

- (*p*(*S* ∪ *i*) − *p*(*S*)) represents the difference in predictions when we include and exclude the feature *i*. For example, in our study, this can be seen as the difference between two CRC probabilities made by two ML models that use two different sets of feature coalitions: with and without a certain bacterial species (e.g., *F. nucleatum*). This value is also called the marginal contribution.
- $\frac{\mid S\mid !\left(n-|S|-1\right)!}{n!}$ represents the weighting for the marginal contributions.
- Finally, the whole equation sums up all possible combinations of weighted marginal contributions, resulting in a SHAP value of the feature *i* for one specific prediction *p*.

SHAPMAT uses the *TreeExplainer* implementation [11] to calculate SHAP values. This implementation is optimized for tree-based models such as random forest and XGBoost, resulting in a faster calculation speed. Since the large feature counts of microbiome data can slow down SHAP calculation, the choice of *TreeExplainer* can help alleviate this issue. Moreover, this implementation is also relevant and beneficial for microbiome research since tree-based models are frequently used in current disease-microbiome association studies [14].

**Fig. 9:**
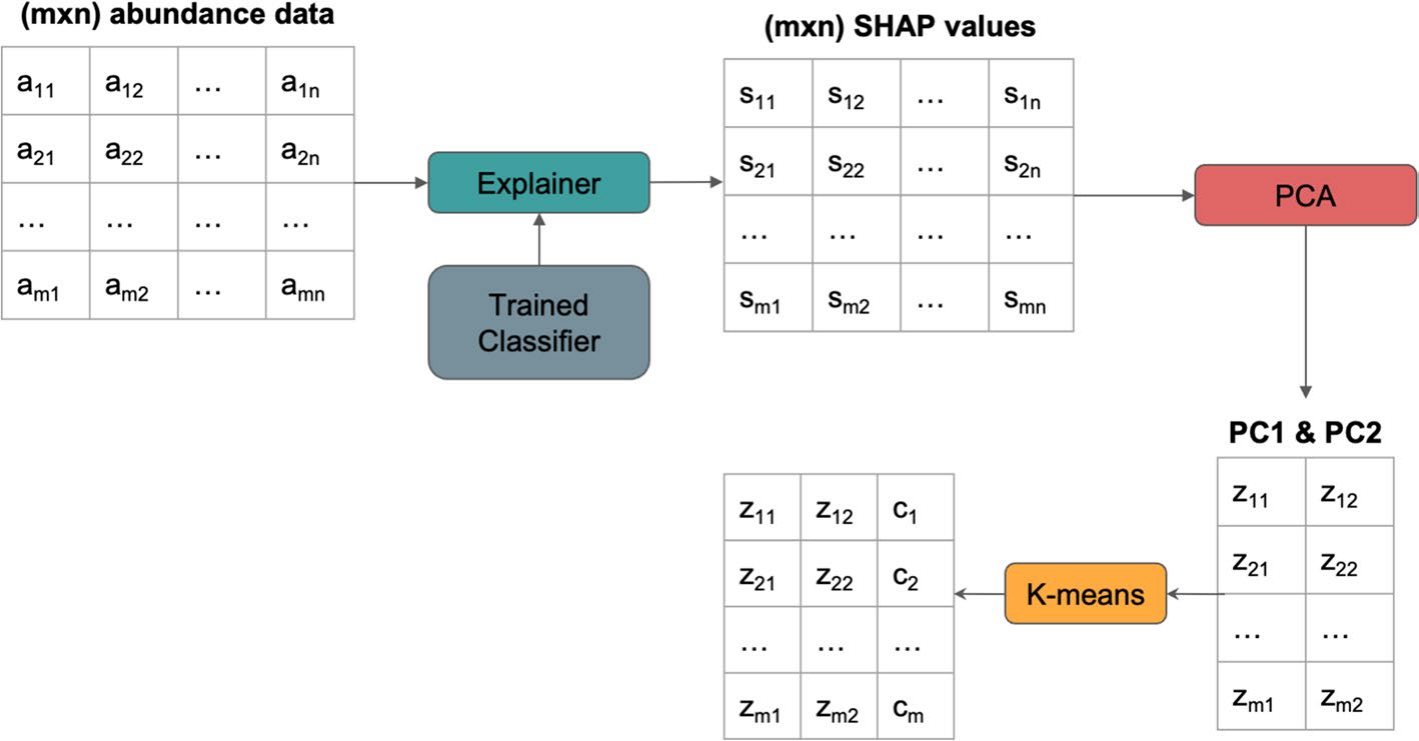
Data flow inside SHAPMAT

Furthermore, to better understand the data flow in SHAPMAT, please refer to Fig. 9. As we can see, if we have a relative abundance table with *m* rows and *n* columns, the resulting SHAP values of this data will also have the same dimension (*m* × *n*). Furthermore, performing PCA to get the first two principal components will reduce the dimension into *m* × 2. It is also possible to specify the number of components other than 2. Finally, *K*-means clustering will assign cluster labels *c*_*i*_ to each sample using the information from the two principal components.

### Statistical analyses

To investigate the dominant bacteria in each cluster, we performed the Kruskal-Wallis *H* test on the relative abundance data across CRC clusters in each dataset. Then, to examine if the resulting bacteria can be potential CRC biomarkers, we conducted the Mann-Whitney *U* test to compare each CRC cluster with the healthy samples. We used 0.05 as the *p*-value threshold for both tests. Please refer to Additional file 1: Tables S1 and S2 for the results.

## Supplementary Information

**Additional file 1: Table S1.** Kruskal Wallis H test Results: dominant bacteria for each cluster. **Table S2.** Mann-Whitney U test Results (CRC cluster vs healthy).

**Additional file 2: Fig. S1.** The Elbow Method Results. **Fig. S2.** Boxplots of Significant Bacteria in Each Cluster.

**Additional file 3.** Review history.

## Data Availability

Source codes for the SHAPMAT library are available on GitHub under the MIT license (https://github.com/ryzary/shapmat) [17]. A version of the source codes used for the analyses in this study is accessible through Zenodo (10.5281/zenodo.7527775) [18]. All the datasets used in this study were obtained using the *curatedMetagenomicData* R package [6]. The following publicly available datasets were used: Yachida et al. [3], Yu et al. [7], Wirbel et al. [5], Zeller et al. [8], Vogtmann et al. [9].
