# Supplementary material for: Leveraging explainable AI for gut microbiome-based colorectal cancer classification
Source: Genome Biol. 2023 Feb 9;24:21. doi: 10.1186/s13059-023-02858-4 (PMC9912568; doi:10.1186/s13059-023-02858-4)
Supplement: Supplementary file 2 — Additional file 2: Fig. S1. The Elbow Method Results. Fig. S2. Boxplots of Significant Bacteria in Each Cluster. [file 13059_2023_2858_MOESM2_ESM.docx]

1. **The Elbow Method Results**

**
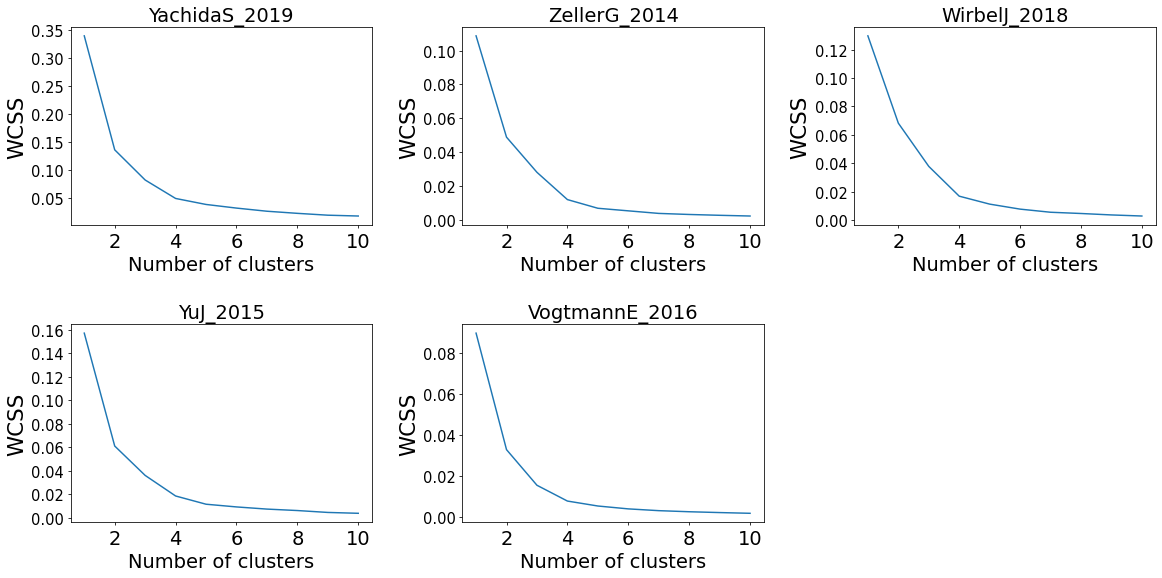
**

***Figure S1. The Elbow method results used to determine the number of CRC clusters in K-means clustering.***

1. **Significant Bacteria in Each Cluster**

**
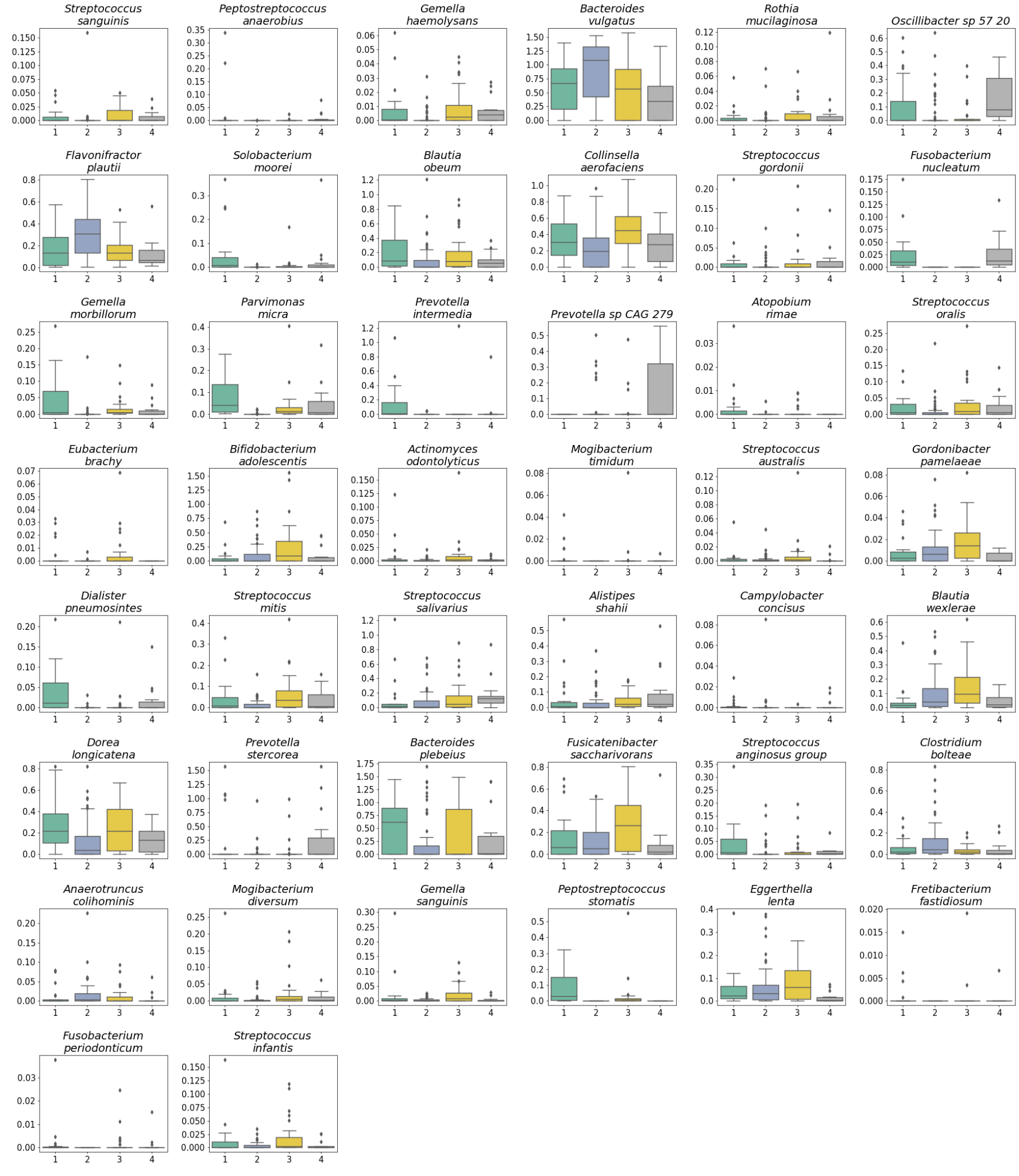
**

***Figure S2. Significant Bacteria in YachidaS_2019 Dataset***

**
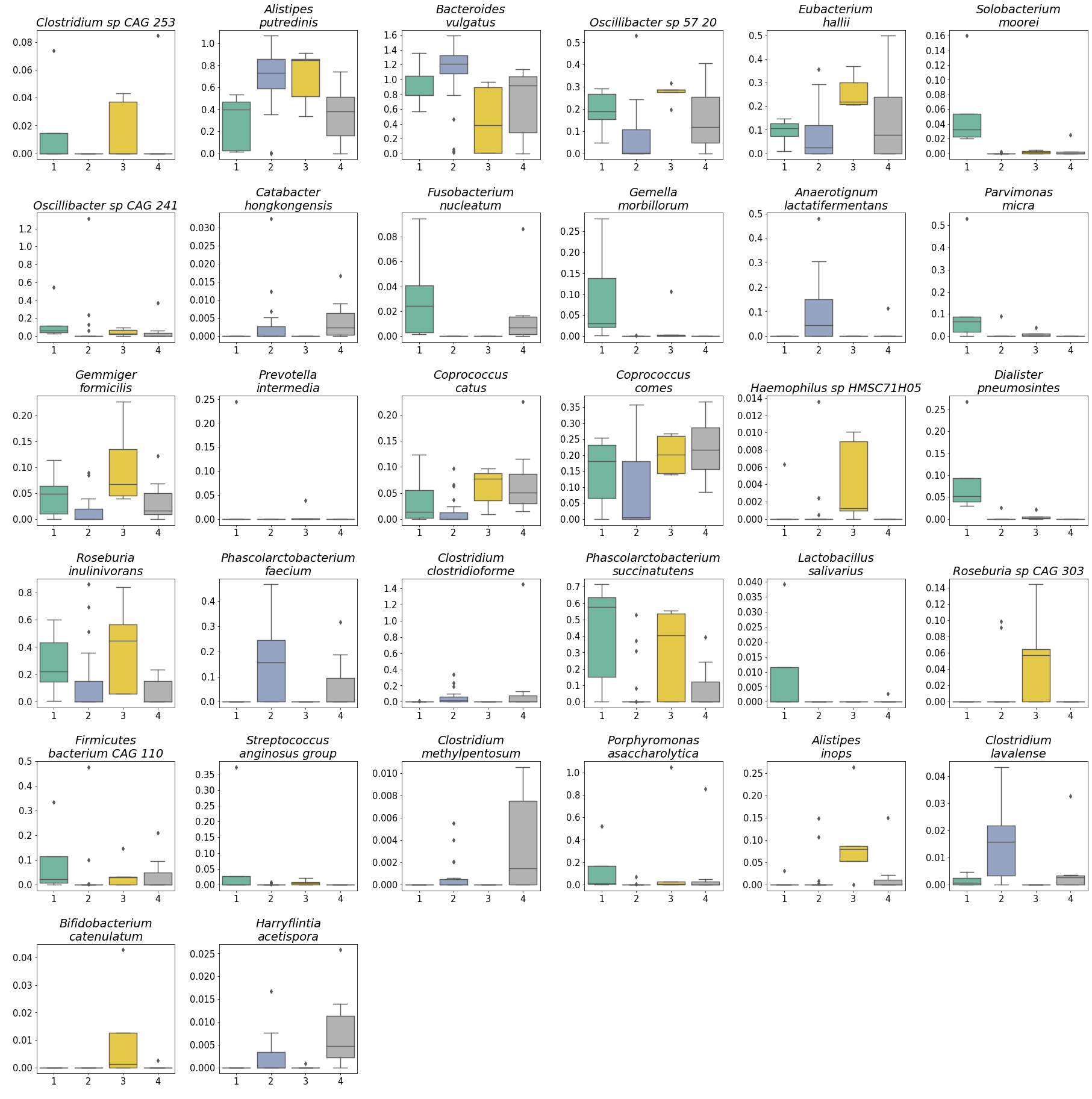
**

***Figure S3. Significant Bacteria in VogtmannE_2016 Dataset***

***
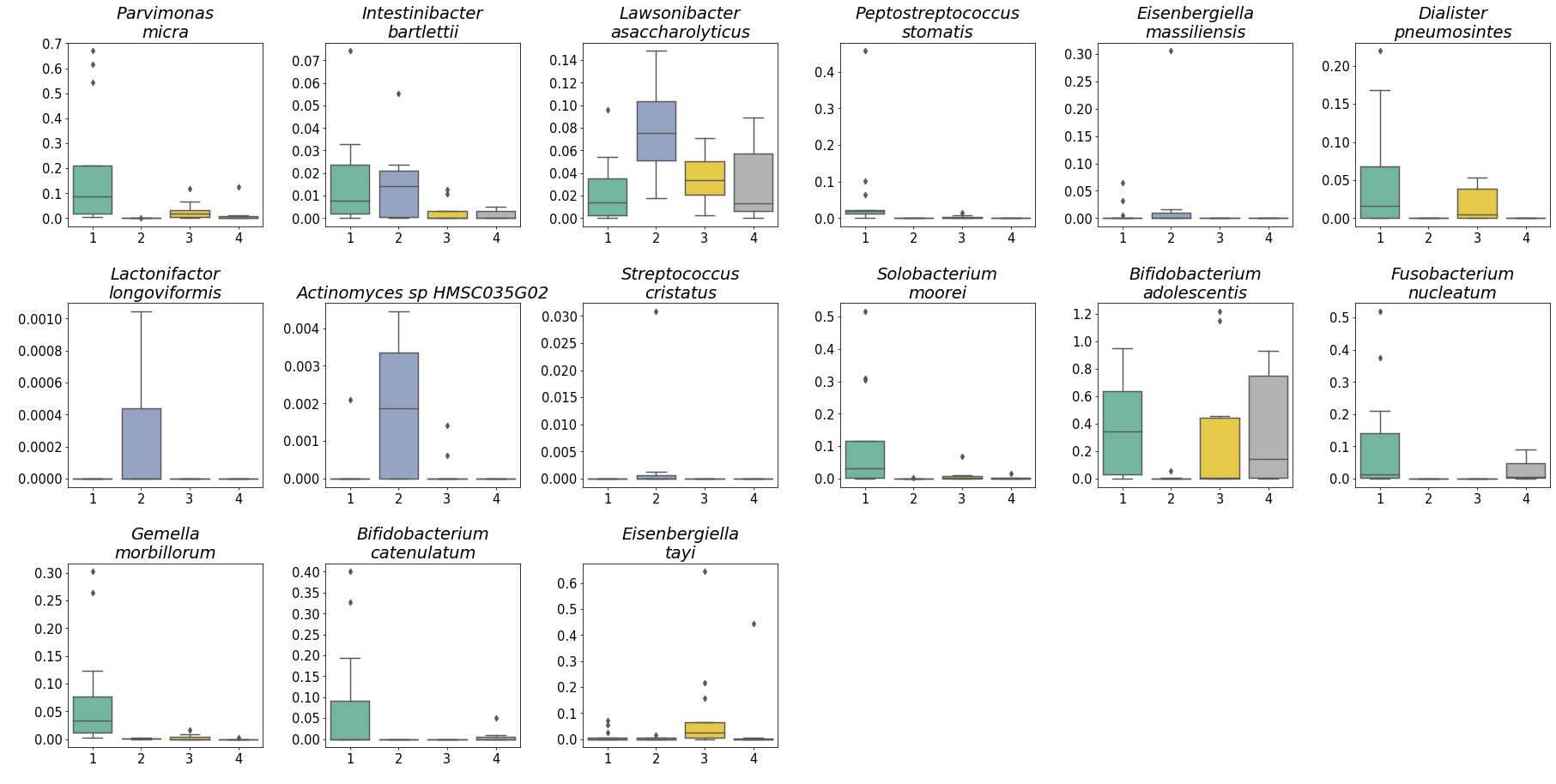
***

***Figure S4. Significant Bacteria in WirbelJ_2018 Dataset***

**
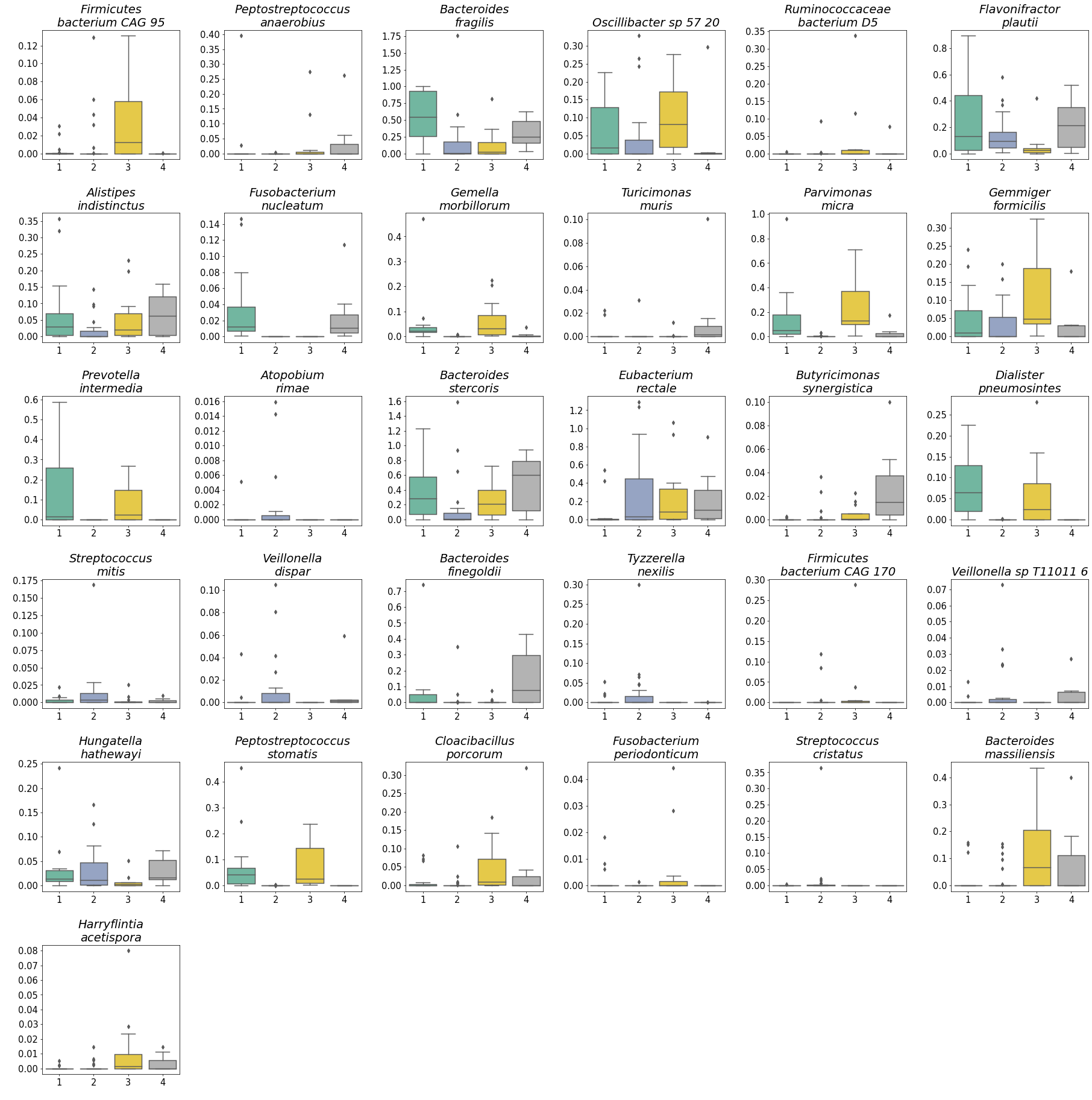
**

***Figure S5. Significant Bacteria in YuJ_2015 Dataset***

**
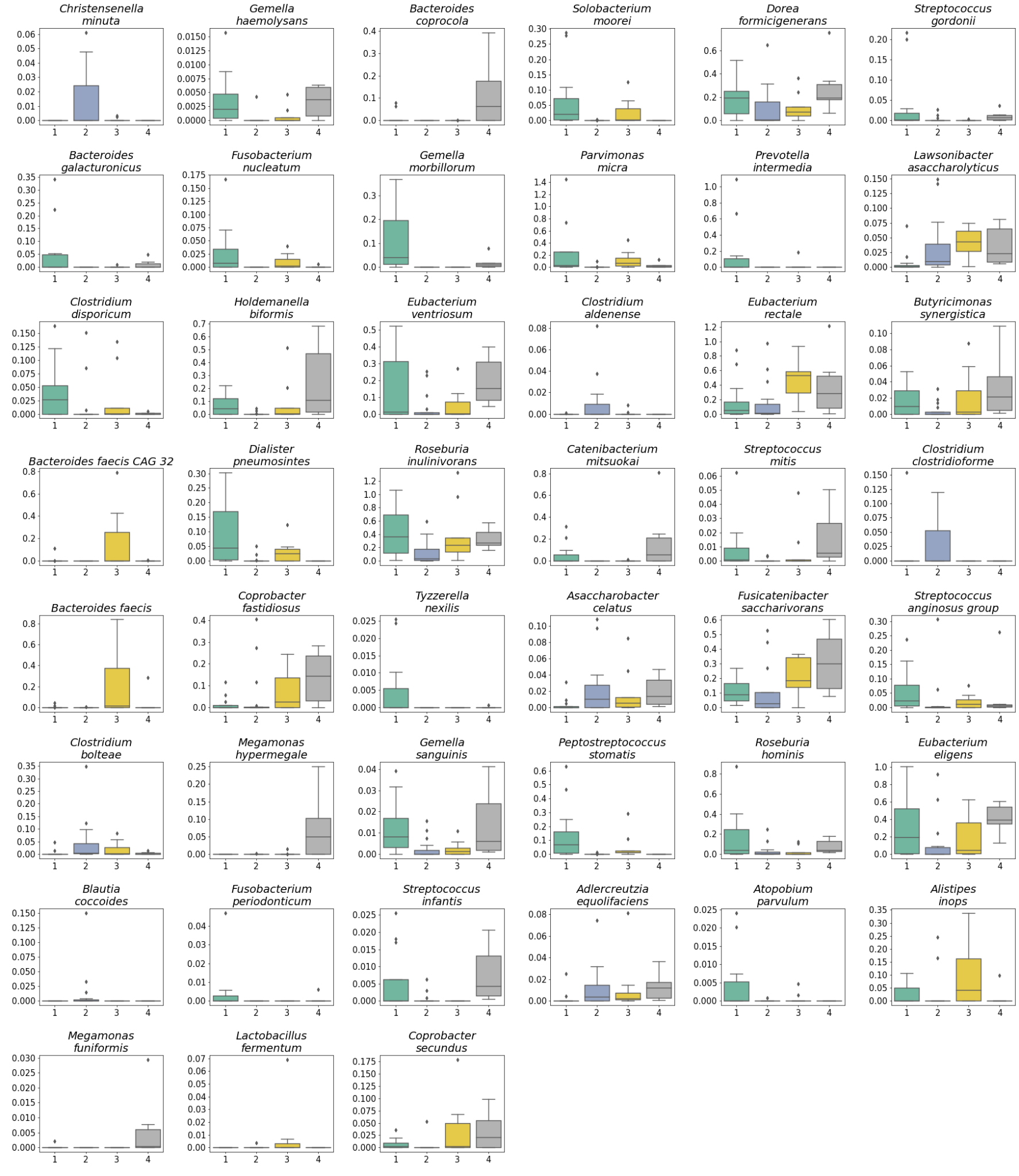
**

***Figure S6. Significant Bacteria in ZellerG_2014 Dataset***
