# Supplementary material for: Leveraging explainable AI for gut microbiome-based colorectal cancer classification
Source: Genome Biol. 2023 Feb 9;24:21. doi: 10.1186/s13059-023-02858-4 (PMC9912568; doi:10.1186/s13059-023-02858-4)
Supplement: Supplementary file 3 — Additional file 3. Review history. [file 13059_2023_2858_MOESM3_ESM.docx]

Review History

**First round of review**

**Reviewer 1**

**Were you able to assess all statistics in the manuscript, including the appropriateness of statistical tests used?** Yes, and I have assessed the statistics in my report.

**Comments to author:**

The manuscript by Ryza Rynazal et al. aim at identifying local contributions of gut bacteria associated with colorectal cancer (CRC). This is accomplished by adopting a local feature contribution method called SHapley Additive exPlanations (SHAP) with the purpose of detect bacteria that are only influential for a subset of individuals. This also enables the identification of subgroups characterized by different biomarkers and associated with different probability values liked to CRC. The methodology is applied on a public available case-control CRC datasets associated with shotgun metagenomic data.

The topic involved in the manuscript is suitable for publication in Genome Biology. The proposed methodology is of potential interest for the microbiome community. However, I have some major comments:
1. One of the key aspects of the manuscript is related to the proposal of the SHAP method. I think the manuscript should be improved in several ways:
- The method is poorly described. A more thorough methodological description should be added in the Methods section of the manuscript;
- I do not see any validation on real and/or synthetic datasets;
- The source code should be provided, along with the scripts necessary to reproduce at least the main/key results of the manuscript
2. The experimental analysis is conducted on a single CRC dataset. Multiple datasets have been published quite recently, please see for example these other two publications:
- Wirbel, Jakob, Paul Theodor Pyl, Ece Kartal, Konrad Zych, Alireza Kashani, Alessio Milanese, Jonas S. Fleck et al. "Meta-analysis of fecal metagenomes reveals global microbial signatures that are specific for colorectal cancer." Nature medicine 25, no. 4 (2019): 679-689.
- Thomas, Andrew Maltez, Paolo Manghi, Francesco Asnicar, Edoardo Pasolli, Federica Armanini, Moreno Zolfo, Francesco Beghini et al. "Metagenomic analysis of colorectal cancer datasets identifies cross-cohort microbial diagnostic signatures and a link with choline degradation." Nature medicine 25, no. 4 (2019): 667-678.
I think the manuscript would largely benefit by the inclusion of such additional datasets to validate/extend findings to independent cohorts.
3. If I understand correctly, the proposed methodology is associated with an AUC of 0.81 in discriminating between CRC and healthy subjects, which is in line with numbers provided by existing methods. Which is the added value of the proposed methodology in terms of prediction capabilities?
4. Which tool did you use to generate taxonomic profiles?

**Reviewer 2**

**Were you able to assess all statistics in the manuscript, including the appropriateness of statistical tests used?** Yes, and I have assessed the statistics in my report.

**Comments to author:**

Rynazal et al. propose the use of subject-specific feature importance values (SHAP) to guide interpretability of existing ML models and showcase the utility of the method by re-analyzing a CRC dataset that leads to the discovery of actionable features and novel CRC subgroups.

The manuscript starts on a really high note motivated by the 'lack of interpretable ML tools for omics-based classification' which I completely agree with where the field should be moving toward. However, the initial enthusiasm dies down quickly as there are several issues that need to be addressed before justifying publication in Genome Biology. My specific comments are summarized as follows:

* As currently presented and evaluated, there is not sufficient evidence that the proposed methodology is not susceptible to false discovery or amenable to the properties of microbiome data such as compositionality and zero-inflation.

* Recent literature (see references below) suggest that the use of SHAP values should be done with caution as a causal interpretation is challenging specially in the presence of multicollinearity which is likely to be exacerbated for microbiome data.

* Given that there is no informed software implementation, it is not clear whether the authors wanted to propose a general methodology to generate individualized feature selection for general omics data or they wanted to simply re-analyze an existing dataset for better biological insights.

The former would require extensive work validated through comprehensive simulation experiments and diverse real data analyses. The latter also requires additional work showcasing the robustness of the method to false positive findings through significance testing with shuffled data. Unfortunately, both these endeavors will require a significant amount of work.

* Given that there are other similar concepts such as LIME, it would be necessary to compare and contrast the SHAP results with that of the LIME to justify the choice of SHAP. What is impact of complex study designs such as longitudinal component and covariates on the SHAP values? Without these additional insights, it's hard to justify the general applicability of the method to other microbiome studies.

* Finally, the lack of a validation cohort without which the generalization of the findings is questionable is the major drawback of the study.

In conclusion, the paper would still be of potential interest to Genome Biology readers if the method is put forward as a general methodology with a software implementation to generate personalized feature selection for general omics data validated through comprehensive simulation experiments and diverse real data analyses. The second best alternative would be conduct additional validation experiments that rule out the effect of false positive findings and showcase the generalizability of the results in an independent validation cohort.


References:

Heskes, Tom, et al. "Causal shapley values: Exploiting causal knowledge to explain individual predictions of complex models." Advances in neural information processing systems 33 (2020): 4778-4789.

Aas, Kjersti, Martin Jullum, and Anders Løland. "Explaining individual predictions when features are dependent: More accurate approximations to Shapley values." Artificial Intelligence 298 (2021): 103502.

Kumar, I. Elizabeth, et al. "Problems with Shapley-value-based explanations as feature importance measures." International Conference on Machine Learning. PMLR, 2020.

**Authors Response**

**Point-by-point responses to the reviewers’ comments:**

We thank all the reviewers for their very useful feedback. Following the editor’s suggestion, we decided to resubmit our manuscript as a ‘Method’ article. The main changes that we added in the revised manuscript are as follows.

1. To validate our approach, we included 4 more datasets from different microbiome studies and conducted similar analyses accordingly.
2. To make our approach usable by other microbiome researchers, we created a software implementation in the form of a python library.

Furthermore, below we provide our responses (in blue) to all the comments and questions from the reviewers.

**Reviewer 1**

1. One of the key aspects of the manuscript is related to the proposal of the SHAP method. I think the manuscript should be improved in several ways:

- The method is poorly described. A more thorough methodological description should be added in the Methods section of the manuscript;

We thank the reviewer for this comment. We agreed that the methodology needed to be elaborated more. We have added a more thorough explanation of SHAP in the Methods section.

- I do not see any validation on real and/or synthetic datasets;

We thank the reviewer for this useful feedback. Following the reviewer’s suggestion, we have included 4 additional validation datasets and analyzed them accordingly.

- The source code should be provided, along with the scripts necessary to reproduce at least the main/key results of the manuscript

We thank the reviewer for pointing this out. The code used for this paper is now accessible through <https://github.com/ryzary/shapmat_paper>. For the software implementation, we created a separate repository containing an installation guide and a tutorial: <https://github.com/ryzary/shapmat>.

2. The experimental analysis is conducted on a single CRC dataset. Multiple datasets have been published quite recently, please see for example these other two publications:

- Wirbel, Jakob, Paul Theodor Pyl, Ece Kartal, Konrad Zych, Alireza Kashani, Alessio Milanese, Jonas S. Fleck et al. "Meta-analysis of fecal metagenomes reveals global microbial signatures that are specific for colorectal cancer." Nature medicine 25, no. 4 (2019): 679-689.

- Thomas, Andrew Maltez, Paolo Manghi, Francesco Asnicar, Edoardo Pasolli, Federica Armanini, Moreno Zolfo, Francesco Beghini et al. "Metagenomic analysis of colorectal cancer datasets identifies cross-cohort microbial diagnostic signatures and a link with choline degradation." Nature medicine 25, no. 4 (2019): 667-678.

I think the manuscript would largely benefit by the inclusion of such additional datasets to validate/extend findings to independent cohorts.

We thank the reviewer for the suggestion. Following the reviewer’s suggestion, we have included 4 additional validation datasets and analyzed them accordingly.

3. If I understand correctly, the proposed methodology is associated with an AUC of 0.81 in discriminating between CRC and healthy subjects, which is in line with numbers provided by existing methods. Which is the added value of the proposed methodology in terms of prediction capabilities?

We thank the reviewer for pointing this out. Our study focuses on leveraging explainable AI for feature contribution analyses; therefore AUC improvement is out of the scope of the current study.

4. Which tool did you use to generate taxonomic profiles?

In the revised manuscript, we utilized the bacterial taxonomic abundance data using the *curatedMetagenomicData* package [1]. These data were obtained using MetaPhlAn3.

**Reviewer 2**

1.As currently presented and evaluated, there is not sufficient evidence that the proposed methodology is not susceptible to false discovery or amenable to the properties of microbiome data such as compositionality and zero-inflation.

We thank the reviewer for this important question. To test if SHAP can be used to analyze microbiome data, we performed additional analyses on the 5 microbiome datasets that were used in the manuscript. These datasets are compositional and zero-inflated. From the table below, we can see that for all dataset, the mean percentage of zeros are above 50 percent, meaning on average, more than half of the data points of a feature are zeros (sparse and zero-inflated).

|  | **Mean Percentage of Zero Abundance** |
| --- | --- |
| **VogtmannE_2016** | 0.561 |
| **WirbelJ_2018** | 0.592 |
| **YachidaS_2019** | 0.653 |
| **YuJ_2015** | 0.576 |
| **ZellerG_2014** | 0.561 |

*Table 1 Mean Percentage of Zero Abundance of Each Bacteria*

For comparison, we also analyzed 3 datasets from studies in other fields that have used SHAP values for feature contribution identification. These datasets have numerical and categorical features that are different from microbiome data. The first study utilizes SHAP to explain ML-based hepatitis predictions [4]. The second study uses SHAP as a feature selection technique for Parkinson’s disease prediction using speed signal data [5]. Finally, the third study explores the potential of SHAP to explain ML models for heart disease diagnosis [6]. All the datasets are publicly available [4-6].

To compare SHAP performance on the microbiome and non-microbiome data, we calculated 3 metrics that have been used in other studies [2-3] for evaluating the performance of explainability methods. The metrics include the following:

1. *Identity*. It states that two identical instances must have identical explanations.
2. *Stability*. It states that instances that are similar must have similar explanations.
3. *Separability*. It states that nonidentical instances must not have identical explanations.

For the microbiome data, we calculated these three metrics using the Leave-One-Dataset-Out (LODO) manner, the same as the main analyses in the manuscript. For the non-microbiome datasets, we conducted 5-fold cross-validation and calculate the SHAP values on the test set only. We used the same procedure as [3] to calculate each metric as follows.

1. To measure the identity metrics, we calculated the distance between two instances and the distance between the corresponding explanations. If the distance between the instances is zero, then the distance between the corresponding explanations must be zero as well.
2. For the stability metrics, we performed K-means clustering on the explanations of all instances. The number of clusters is equal to the number of classes. For all instances, we compared the predicted cluster labels of the explanations with the instances’ predicted class. If these two predictions matched, then stability is satisfied.
3. Finally for the separability metrics, we selected a subset of data that has no duplicates. Then, we obtained their explanations. For every instance in this subset, we compared its explanation to the explanations of the remaining instances. If there is no duplicate, then separability is satisfied.

The result is shown below. The numbers show the percentage of instances that satisfy the metrics.

|  | Identity | Separability | Stability |
| --- | --- | --- | --- |
| **YachidaS_2019** | 100% | 100% | 74.10% |
| **ZellerG_2014** | 100% | 100% | 62.30% |
| **WirbelJ_2018** | 100% | 100% | 75.20% |
| **YuJ_2015** | 100% | 100% | 65.60% |
| **VogtmannE_2016** | 100% | 100% | 63.50% |
|  |  |  |  |
| **Hepatitis Dataset** | 100% | 100% | 77.65% |
| **Parkinson's Disease Dataset** | 100% | 100% | 80.55% |
| **Heart Disease Dataset** | 100% | 100% | 79.20% |

The identity scores for all the microbiome data are 100%, suggesting that SHAP was able to generate the same explanations for the same instances in the microbiome data, even when the data are compositional, sparse, and the zero-inflated. In addition, the separability scores for all the microbiome datasets are also 100%, showing perfect separation between nonidentical instances. The non-microbiome data also showed the same scores for these two metrics. Furthermore, as for the stability metrics, the microbiome data show relatively lower scores than the non-microbiome data. However, this also varies for different microbiome datasets. The ZellerG_2014 dataset’s stability score is the lowest (62.30%) and the WirbelJ_2018 dataset’s stability score is the highest (75.20%).

Based on these results, in comparison to studies in other fields that utilized SHAP for feature contribution identification [4-6], our analyses indicate that SHAP still shows a comparable performance when applied to the microbiome data despite the challenging properties of the data being compositional, sparse, and zero-inflated. Therefore, we believe that our proposed methodology is usable for microbiome data analyses.

2.Recent literature suggest that the use of SHAP values should be done with caution as a causal interpretation is challenging specially in the presence of multicollinearity which is likely to be exacerbated for microbiome data.

We thank the reviewer for this excellent point. We fully agree with this limitation of SHAP in inferring causal interpretations. SHAP is often used to explain ML model predictions especially black box models such as Random Forest and XGBoost. These models are not inherently causal to begin with, therefore the produced explanations would contain information of the *correlations* learned by the model, not *causation*. Similarly, correlation bias induced by multicollinearity is caused by the way ML models are trained and not directly due to the SHAP method itself.

While these challenges limit the scope of this manuscript, we believe that our methodology is still very useful for the microbiome community since many microbiome studies are still in the stage of investigating microbiota-phenotype associations (correlations) where ML models are frequently used.

3. Given that there is no informed software implementation, it is not clear whether the authors wanted to propose a general methodology to generate individualized feature selection for general omics data or they wanted to simply re-analyze an existing dataset for better biological insights.

We thank the reviewer for this comment. As we resubmitted the manuscript as a ‘Method’ article, we intended to propose methodologies that leverage SHAP for microbiome data analyses that include feature selection (global and local) and subtyping. We used existing datasets to show the capabilities of these methodologies.

We also included a software implementation as a python library called SHAPMAT (SHAP-based Microbiome Analysis Tool). This tool can be used by microbiome researchers to use SHAP for feature importance analyses and disease subtyping/clustering.

4.Given that there are other similar concepts such as LIME, it would be necessary to compare and contrast the SHAP results with that of the LIME to justify the choice of SHAP. What is impact of complex study designs such as longitudinal component and covariates on the SHAP values? Without these additional insights, it's hard to justify the general applicability of the method to other microbiome studies.

We thank the reviewer for this excellent question. Currently, we focus our method on cross-sectional studies. Therefore, longitudinal components of data are out of the scope of the current study. As for the effect of covariates, since SHAP is a method to explain an ML prediction, it will not be able to quantify the effect of covariates such as age or gender unless we include them as features for training the ML model.

To answer the question about the justification for choosing SHAP over LIME, we conducted the same analyses as our answer to the first question. We calculated the identity, separability, and stability metrics of SHAP and LIME using the microbiome datasets. The results are summarized below.

|  | **Identity** | | **Separability** | | **Stability** | |
| --- | --- | --- | --- | --- | --- | --- |
|  | SHAP | LIME | SHAP | LIME | SHAP | LIME |
| **YachidaS_2019** | 100% | 0% | 100% | 100% | 74.10% | 70% |
| **ZellerG_2014** | 100% | 0% | 100% | 100% | 62.30% | 69.30% |
| **WirbelJ_2018** | 100% | 0% | 100% | 100% | 75.20% | 77.60% |
| **YuJ_2015** | 100% | 0% | 100% | 100% | 65.60% | 78.10% |
| **VogtmannE_2016** | 100% | 0% | 100% | 100% | 63.50% | 63.50% |

In all datasets, SHAP shows 100% score for the identity metrics whereas LIME shows 0%. This suggests that SHAP constantly generates the same explanations for the same instances while LIME explanations are always changing even for the same instances. As for the separability metrics, both methods show the same scores across all datasets. Finally, for the stability metrics, LIME and SHAP show comparable performances where the mean stability of SHAP and LIME across all datasets are 68.4% and 71.7%, respectively.

While the separability and stability scores are comparable between the two methods, the low identity score for LIME (0% in all datasets) shows that this method cannot generate reproducible explanations even for the same instances. This inconsistency is not desirable especially when we want to produce local explanations for each instance, which is one of the objectives of our study. Based on these results, we decided to use SHAP over LIME.

5.Finally, the lack of a validation cohort without which the generalization of the findings is questionable is the major drawback of the study.

We thank the reviewer for this useful feedback. Following the reviewer’s suggestion, we have included 4 additional validation datasets and analyzed them accordingly.

**References**

1. Pasolli, E., Schiffer, L., Manghi, P., Renson, A., Obenchain, V., Truong, D. T., Beghini, F., Malik, F., Ramos, M., Dowd, J. B., Huttenhower, C., Morgan, M., Segata, N., & Waldron, L. (2017, November). Accessible, curated metagenomic data through ExperimentHub. Nature Methods, 14(11), 1023–1024. <https://doi.org/10.1038/nmeth.4468>
1. Honegger, M. (2018, August 15). Shedding Light on Black Box Machine Learning Algorithms: Development of an Axiomatic Framework to Assess the Quality of Methods that Explain Individual Predictions. Arxiv. <https://doi.org/10.48550/ARXIV.1808.05054>
2. ElShawi, R., Sherif, Y., Al‐Mallah, M., & Sakr, S. (2020, November 24). Interpretability in healthcare: A comparative study of local machine learning interpretability techniques. Computational Intelligence, 37(4), 1633–1650. <https://doi.org/10.1111/coin.12410>
3. Peng, J., Zou, K., Zhou, M., Teng, Y., Zhu, X., Zhang, F., & Xu, J. (2021). An Explainable Artificial Intelligence Framework for the Deterioration Risk Prediction of Hepatitis Patients. Journal of Medical Systems, 45(5). <https://doi.org/10.1007/s10916-021-01736-5>
4. Liu, Y., Liu, Z., Luo, X., & Zhao, H. (2022). Diagnosis of Parkinson’s disease based on SHAP value feature selection. Biocybernetics and Biomedical Engineering, 42(3), 856–869. <https://doi.org/10.1016/j.bbe.2022.06.007>
5. Rajendran, P., Haw, S. C., & Naveen, P. (2021). Classification of Heart Disease Using Machine Learning Techniques. 2021 5th International Conference on Digital Technology in Education. <https://doi.org/10.1145/3488466.3488482>

**Second round of review**

**Reviewer 1**

The manuscript has been improved in this revised version. Authors have answered positively to my previous comments and modified the manuscript according to them.

**Reviewer 2**

The authors have done a decent job revising the earlier manuscript. Most of the major concerns raised in the first round review have been resolved. The added real data analysis show some very convincing results. Overall, the paper is now in a very good condition to be published.
